# Neglected Tropical Diseases: A Chemoinformatics Approach for the Use of Biodiversity in Anti-Trypanosomatid Drug Discovery

**DOI:** 10.3390/biom14081033

**Published:** 2024-08-20

**Authors:** Marilia Valli, Thiago H. Döring, Edgard Marx, Leonardo L. G. Ferreira, José L. Medina-Franco, Adriano D. Andricopulo

**Affiliations:** 1 Laboratory of Medicinal and Computational Chemistry (LQMC), Center for Research and Innovation in Biodiversity and Drug Discovery (CIBFar), Institute of Physics of Sao Carlos, University of Sao Paulo (USP), Av. Joao Dagnone, n° 1100, Sao Carlos 13563-120, SP, Brazil; thiago.doring@ufsc.br (T.H.D.); leonardo@ifsc.usp.br (L.L.G.F.); 2 School of Pharmaceutical Sciences of Ribeirao Preto (FCFRP), University of Sao Paulo (USP), Avenida Professor Doutor Zeferino Vaz, s/n, Ribeirao Preto 14040-903, SP, Brazil; 3 Department of Exact Sciences and Education (CEE), School of Technology, Exact Sciences and Education (CTE), Federal University of Santa Catarina (UFSC), Blumenau 89036-256, SC, Brazil; 4 Agile Knowledge Engineering and Semantic Web (AKSW), Institute of Computer Science, Leipzig University of Applied Sciences (HTWK), 04109 Leipzig, Germany; edgard.marx@htwk-leipzig.de; 5 DIFACQUIM Research Group, Department of Pharmacy, School of Chemistry, Universidad Nacional Autonoma de Mexico (UNAM), Avenida Universidad 3000, Mexico City 04510, Mexico; medinajl@unam.mx

**Keywords:** natural products, neglected tropical diseases, drug discovery, chagas disease, leishmaniasis, human African trypanosomiasis

## Abstract

The development of new treatments for neglected tropical diseases (NTDs) remains a major challenge in the 21st century. In most cases, the available drugs are obsolete and have limitations in terms of efficacy and safety. The situation becomes even more complex when considering the low number of new chemical entities (NCEs) currently in use in advanced clinical trials for most of these diseases. Natural products (NPs) are valuable sources of hits and lead compounds with privileged scaffolds for the discovery of new bioactive molecules. Considering the relevance of biodiversity for drug discovery, a chemoinformatics analysis was conducted on a compound dataset of NPs with anti-trypanosomatid activity reported in 497 research articles from 2019 to 2024. Structures corresponding to different metabolic classes were identified, including terpenoids, benzoic acids, benzenoids, steroids, alkaloids, phenylpropanoids, peptides, flavonoids, polyketides, lignans, cytochalasins, and naphthoquinones. This unique collection of NPs occupies regions of the chemical space with drug-like properties that are relevant to anti-trypanosomatid drug discovery. The gathered information greatly enhanced our understanding of biologically relevant chemical classes, structural features, and physicochemical properties. These results can be useful in guiding future medicinal chemistry efforts for the development of NP-inspired NCEs to treat NTDs caused by trypanosomatid parasites.

## 1. Introduction

Neglected tropical diseases (NTDs) are a group of twenty diseases of poverty that impose a devastating human, social, and economic burden on more than one billion people in tropical and subtropical areas of the world [1]. The World Health Organization (WHO) 2021–2030 Road Map comprises global targets and indicators to prevent, control, eliminate, or eradicate NTDs by 2030, see ref. [2], as well as cross-cutting targets aligned with the United Nations Sustainable Development Goals (SDGs) [3].

According to the 2023 Global Report on Neglected Tropical Diseases 2023 (WHO) [4], noteworthy progress has been made since the launch of the road map. For example, 47 countries had eliminated at least one NTD by the end of December 2022. The number of people requiring interventions against NTDs has decreased by 25% over the past decade, with a reduction of 81 million people between 2020 and 2021 alone, from 1.734 billion to 1.653 billion. Nonetheless, many difficulties in achieving the targets for 2030, in addition to the COVID-19 pandemic, have revealed the scale of the task still ahead. The 2021–2022 period saw several outbreaks of NTDs, including dengue, chikungunya, leishmaniasis, Chagas disease, and scabies. The COVID-19 pandemic led to a substantial reduction in the number of people receiving interventions against NTDs. In 2020, only 798 million individuals had received treatment for at least one NTD, a reduction of 34% compared with 2019, when this figure amounted to 1.207 billion. In 2021, 90 million more people were treated, bringing the total to 888 million (+11%). Although the positive trend registered in 2021 is likely to continue, the difference from the pre-COVID-19 era is substantial, when more than one billion people were treated every year for four consecutive years between 2016 and 2019 [4].

Scientists working with NTDs are confronted with a long-standing challenge: the current treatments available have limitations in terms of safety and efficacy, among others; and inconceivably, from the 1970s to 2023, no New Chemical Entities (NCEs) were developed for this group of diseases that account for about 11% of the total disease burden in the world [5]. In this period, only new formulations or repositioned compounds were approved for these 20 conditions. In the 21st century alone, miltefosine was repurposed for leishmaniasis (2014), moxidectin for onchocerciasis (2018), fexinidazole for human African trypanosomiasis (HAT, 2021), and a pediatric formulation of benznidazole was approved for Chagas disease (2017) [1,4].

**Figure 1:**
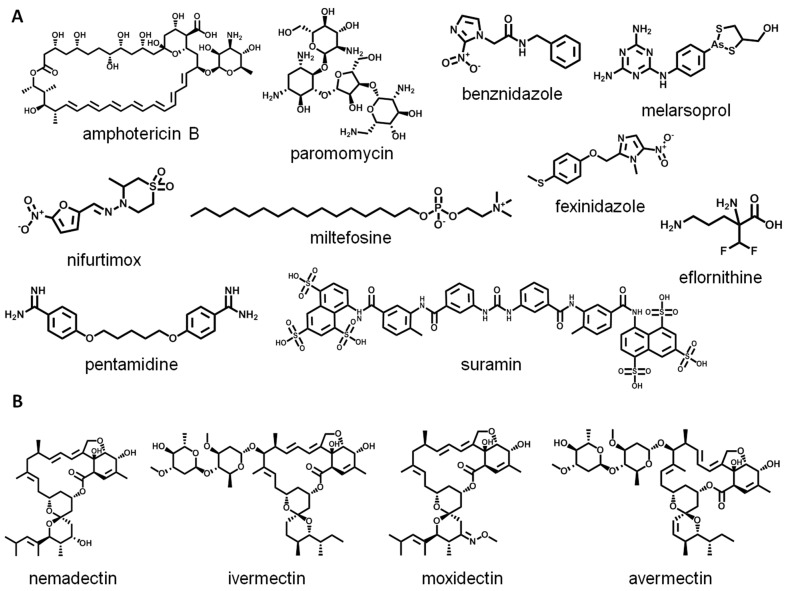
Drugs used for NTDs: (**A**) drugs for trypanosomatid diseases, (**B**) drugs for other NTDs.

Although battling NTDs should be a priority for humanity and sustainability, there is a clear lack of investment in research and development (R&D) programs, and the NTD market is unattractive to the pharmaceutical industry [4,5,6]. Therefore, it is of great importance to focus on the discovery of NCEs for the treatment of NTDs. Natural products (NPs) are valuable sources for the development of drugs for a variety of human diseases. This includes NTDs, such as the anti-leishmanial agent amphotericin B (Figure 1A), extracted from *Streptomyces noclosus* and primarily used to treat fungal infections. The antimicrobial aminoglycoside paromomycin (Figure 1A), produced by *Streptomyces krestomuceticus*, is used to treat leishmaniasis. Moxidectin (Figure 1B), employed to treat onchocerciasis, is obtained from the modification of the NP nemadectin (Figure 1B), which was isolated from *Streptomyces cyaneogriseus*. Ivermectin (Figure 1B), used for the treatment of onchocerciasis, lymphatic filariasis, scabies, and other ectoparasitoses, is a dihydro analogue of the macrocyclic lactone avermectin (Figure 1B), whose analogues were obtained from *Streptomyces avermitilis*, an actinomycete present in soil.

NPs have a long history of achievements in the early stages of R&D initiatives as a source of new hits and inspiration for new lead compounds with privileged drug-like properties for NTD drug discovery. In this work, we concentrate our efforts on NTDs caused by trypanosomatid parasites, Chagas disease, HAT, and leishmaniasis, in which NCEs are needed to enable new generations of therapies to revolutionize the clinical treatment of these diseases, and to save millions of lives [7]. Chagas disease, caused by the parasite *Trypanosoma cruzi*, is endemic in 21 Latin American countries [8]. There are 6–7 million people infected worldwide, with another 75 million at risk of contamination. Only two old nitro-heterocyclic drugs—benznidazole and nifurtimox (Figure 1A)—are available, and both have several limitations. Leishmaniasis, caused by more than 20 species of *Leishmania* sp., affects 700,000 to 1 million people every year, and its visceral form is fatal if left untreated in over 95% of cases, with about 50,000–90,000 new cases each year [5]. The existing drugs have variable efficacy and serious toxicities—amphotericin B, pentavalent antimonials, and paromomycin—and only one, miltefosine (Figure 1A), is administered orally, whereas the others are given by intravenous or intramuscular injections. HAT, caused by *Trypanosoma brucei gambiense* (g-HAT) and *T. b. rhodesiense* (r-HAT), is endemic in sub-Saharan Africa. Seventy million people are at risk of infection, [9] and the therapies available are based on highly toxic compounds: melarsoprol, eflornithine, suramin, pentamidine, and nifurtimox (Figure 1A). Fexinidazole was introduced in 2021 as the first effective oral monotherapy against g-HAT (Figure 1A).

**Figure 2:**
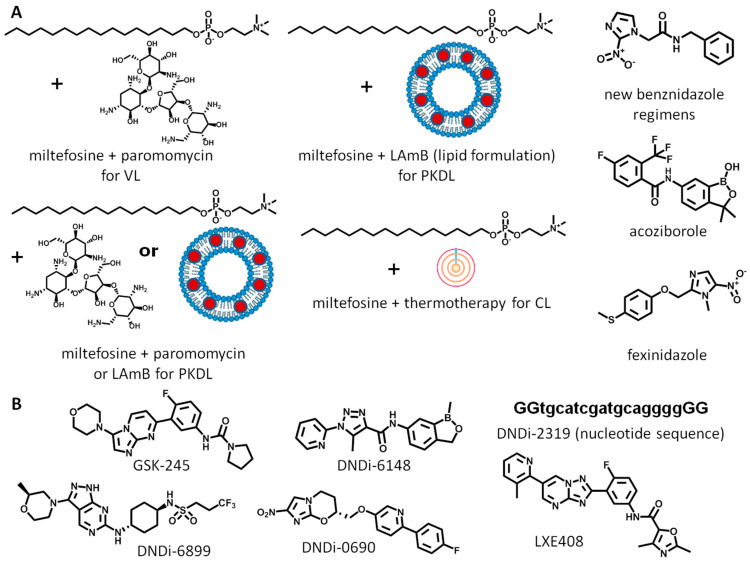
Current clinical pipeline for Chagas disease, leishmaniasis, and HAT: (**A**) advanced clinical trials, (**B**) early stages of clinical development. DNDi-2319 uppercase: phosphothionate bases; lowercase: phosphodiester bases. VL: visceral leishmaniasis; PKDL: post-kala-azar dermal leishmaniasis; CL: cutaneous leishmaniasis; LAmB: liposomal amphotericin B.

The current clinical pipeline (DNDi R&D portfolio, 2023) for anti-trypanosomatid drug discovery (Figure 2) [10], focusing on more advanced clinical trials (phase IIb/III and registration), is dominated by new formulations, new regimens, or combinations of old drugs (Figure 2) for leishmaniasis. The number of compounds is modest and represents a well-known repertory of unsatisfactory drugs (amphotericin B, paromomycin, miltefosine, and fexinidazole; Figure 1A). For Chagas disease, only new regimens of benznidazole are under consideration (Figure 2A). For HAT, orally active acoziborole is in phase IIb/III (Figure 2A). Under registration, there is only a drug combination (miltefosine + paromomycin) for visceral leishmaniasis, and fexinidazole for HAT (Figure 2A). In the early stages of the clinical pipeline—phase I and phase IIa/proof-of-concept—there are a few NCE candidates in clinical development (Figure 2B) [10]. The situation is critical for Chagas disease; only one compound is in phase I, a benzoxaborole derivative (DNDI-6148, CPSF3 inhibitor) (Figure 2B). For leishmaniasis, six candidates are under investigation. Five candidates are in phase I: DNDI-0690 (bioactivation by NTR2), GSK-245 (proteasome inhibitor), DNDi-6148 (CPSF3 inhibitor), DNDI-6899 (CRK12 inhibitor), and DNDi-2319 (oligonucleotide). In phase II, there is only a proteasome inhibitor (LXE408) (Figure 2B). For HAT, no compounds are under consideration in the early stages.

Chemoinformatics have played an important role in the hit identification and hit-to-lead stages of drug discovery, allowing us to focus on privileged chemical scaffolds (lead compounds) that exhibit promising drug-like properties [11]. In this study, we examined the literature from 2019 to March 2024 to identify NP compounds with promising anti-trypanosomatid activity. As part of the literature survey, we created a database and analyzed the structural content, distribution in chemical space, and determined several molecular and physicochemical properties of pharmaceutical interest using computational tools. We also used chemoinformatic approaches to reveal important insights into the understanding of the chemical classes, molecular scaffolds, and corresponding drug-like properties of small-molecule NPs. The findings of this study can be useful in guiding future medicinal chemistry efforts to develop NP-based NCEs for NTDs caused by trypanosomatid parasites.

## 2. Materials and Methods

**Figure 3:**
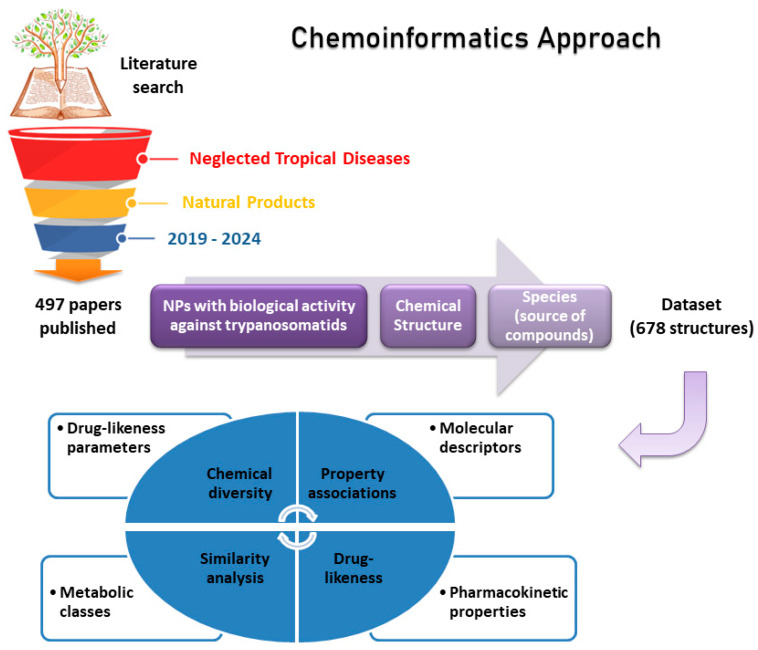
Strategy used to build the dataset used in this study.

**Literature search.** The literature search was performed on 2 April 2024 with the keywords described in Table 1 to construct the dataset used in this study. With the aim of conducting an extensive search, we used the SciFinder-n (Chemical Abstracts Service, Columbus, OH, USA) and Web of Science (Clarivate, London, UK) platforms [12,13]. A total of 497 papers published from 2019 to March 2024 were selected. The papers were individually analyzed to extract information on the bioactive compounds tested against *T. cruzi*, *T. brucei*, or eight *Leishmania* species for the creation of the dataset (Figure 3).

**Dataset.** The complete compound dataset used in this study is available in the Supporting Information (Supporting Information). The literature search was manually analyzed to identify NPs reported to have biological activity (IC_50_ determined or percentage of inhibition greater than 50%) against trypanosomatid parasites. Using these criteria, information on 678 NPs was collected (Supporting Information).

**Molecular descriptors and pharmacokinetic properties.** Molecular descriptors, pharmacokinetic properties, and drug-likeness parameters were computed using the SwissADME platform (University of Lausanne and the SIB Swiss Institute of Bioinformatics, Lausanne, Switzerland) [14]. Descriptors for the ring count analyses were calculated using the QikProp module in Maestro v. 11.2.013 (Schrödinger, New York, NY, USA). The clogP values for the n-octanol/water system was calculated using the implicit logP method provided by the SwissADME platform (University of Lausanne and the SIB Swiss Institute of Bioinformatics, Lausanne, Switzerland). Metabolic classes were determined with the aid of Classyfire v. 1.0 (Edmonton, AB, Canada) [15]. The calculated data can be found in the Supporting Information.

**Structural fingerprint.** For the structural similarity analyses, the Canvas Fingerprint Similarity module was used (Maestro v. 11.2.013, Schrödinger, New York, NY, USA). The linear fingerprint type was used. The cluster was built using 64-bit precision. The atom typing scheme was distinguished by ring size, aromaticity, HBA/HBD, ionization potential, and whether the atom is terminal or halogen. Bonds were distinguished by bond order. The similarity metric applied was calculated using the Tanimoto coefficient [16] and the linkage average method.

**Molecular properties of chemical space and principal component analysis.** Four molecular properties of pharmaceutical interest were computed in DataWarrior (v. 5.5.0, Actelion/Idorsia Pharmaceuticals Ltd., Allschwil, Switzerland) [17]: nRotB, HBA, HBD, and MW (PC1 = 64.649%; PC2 = 20.397%; PC3 = 9.756%). The linear correlation between descriptors was performed using the Bravais–Pearson coefficient [18]. Candlestick charts, means, medians, and quartiles were calculated using DataWarrior (v. 5.5.0, Actelion/Idorsia Pharmaceuticals Ltd., Allschwil, Switzerland).

## 3. Results and Discussion

### 3.1. Annotated Compound Database

From 2019 to March 2024, 497 research articles reported NPs with anti-trypanosomatid activity isolated from plants (73.0%), marine organisms (12.1%), fungi (4.9%), bacteria (3.1%), and animals (1.5%), as well as NP derivatives (1.9%) and compounds from NP databases (3.5%). The 497 articles selected for this study were published in the following years: 169 (24.9%) were published in 2019, 147 (21.7%) in 2020, 127 (18.7%) in 2021, 116 (17.1%) in 2022, 105 (15.5%) in 2023 and 14 (2.1%) until March 2024. The chemical and biological data collected were analysed, and a comprehensive compound dataset was generated for a unique set of 678 small-molecule bioactive NPs. Structures belonging to different metabolic classes were identified. Terpenoids represent the largest class of compounds in the dataset, with 207 structurally diverse compounds (30.5%), which is unsurprising due to their high abundance among NPs. Benzoic acids and benzenoids represent 19.5% of the dataset, with 132 structures. Furthermore, the dataset includes 63 alkaloids (9.3%), 63 polyketides (9.3%), 58 phenylpropanoids (8.6%), 44 flavonoids (6.5%), 34 peptides/peptide mimetics (5.0%), 25 lignans/neolignans (3.7%), 10 naphthoquinones (1.5%), 4 cytochalasins (0.6%), 1 xanthone (0.1%), and 1 tannin (0.1%). The remaining 32 compounds (4.7%) do not belong to any of the mentioned classes or to a single chemical class. The complete database, annotated with the chemical and biological data, is available in the Supporting Information.

### 3.2. Ring Content, Structural Alerts, and Synthetic Accessibility

**Figure 4:**
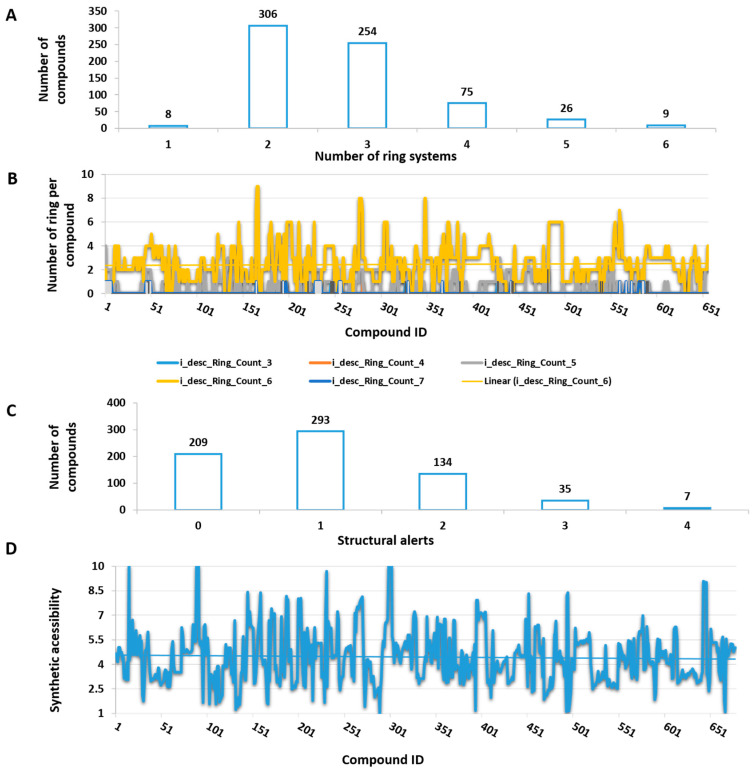
Profile of the dataset with 678 compounds regarding ring count, structural alerts, and calculated synthetic accessibility: (**A**) ring count considering any ring size, (**B**) number of rings in each structure of the dataset, (**C**) Brenk structural alerts, (**D**) synthetic accessibility scores using the SwissADME webserver (University of Lausanne and the SIB Swiss Institute of Bioinformatics, Lausanne, Switzerland).

A chemoinformatic study was conducted to investigate the chemical space coverage of the dataset and to explore the biologically relevant molecular diversity for anti-trypanosomatid drug discovery. Initially, the 678 dataset compounds were grouped by ring count (Figure 4A). Most chemical structures (83% of the dataset) possess two or three ring systems, with a predominance of five- or six-membered rings (Figure 4B). Among those molecules with six-membered rings, for example, are terpenes, steroids, and flavonoids containing three rings, whereas aromatic derivatives, phenylpropanoids, and lignans bear two rings.

Next, the dataset was evaluated for the identification of structural alerts (based on Brenk filters) [19] for potentially toxic or unstable chemical moieties (Figure 4C). Most of the dataset compounds present a good drug-like profile: 502 compounds (74%) have one or no alerts; 134 (20%), 35 (5%), and 7 (1%) compounds, respectively, present two, three, and four alerts. Moreover, the synthetic accessibility of the dataset compounds was analyzed by scores varying from 1 (easiest) to 10 (most difficult), using the molecular fingerprint (FP) approach and the metric system implemented in the SwissADME webserver (University of Lausanne and the SIB Swiss Institute of Bioinformatics, Lausanne, Switzerland) (Figure 4D) [14]. A tendency line shows that, on average, the dataset features a synthetic accessibility score between 4 and 5, which indicates an acceptable number of reaction steps to synthesize the target NP compounds. The FP method is based on the construction of a sequence of bits that determines the presence or absence of a chemical descriptor in a molecule. The final model was constructed using 1024 fragments and trained with more than 12 million structures. The model yielded a correlation coefficient value (*r*) of 0.94.

### 3.3. Drug-Likeness

**Figure 5:**
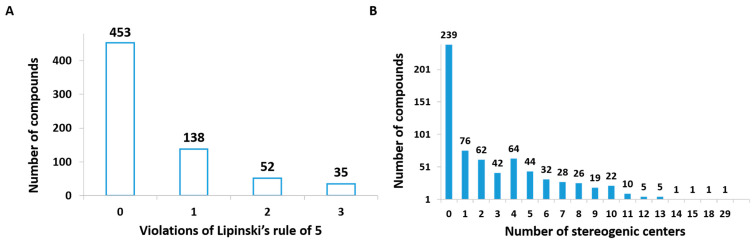
Investigation of molecular properties and structural complexity of the dataset compounds: (**A**) violations of Lipinski’s rule of five, (**B**) number of stereogenic centers.

As stated by Lipinski’s rule of five (Ro5), oral drug-like compounds with good solubility and permeability should have no more than 5 hydrogen-bond donors, no more than 10 hydrogen-bond acceptors, a molecular weight (MW) no greater than 500 Da, and a calculated n-octanol/water partition coefficient (clogP) no greater than 5 [20]. With no more than one violation of the Ro5 criteria, 591 (87%) compounds of the dataset have high potential for oral bioavailability (Figure 5A). Although there are other compound filters based on different combinations of descriptors in use today, the use of the most traditional and well-known group of Lipinski’s filters is of particular interest.

### 3.4. Stereogenic Centers

Bioactive NPs are usually associated with complex structures with high MW, moving way beyond small molecules that fall within the Ro5. As discussed in the previous section, our results indicate that 87.2% of the NPs reported in the last five years (2019 to March 2024) represent small molecules with drug-like properties for anti-trypanosomatid drug discovery. Another important finding is related to the number of stereogenic centers present in the dataset structures. Compounds with multiple chiral centers are avoided in NTD drug discovery programs due to their synthetic complexity and significant challenges for the generation of analogue series for structure–activity relationship (SAR) studies [21]. Among the NPs of the dataset, 35.3% (239 compounds) do not present stereogenic centers, 11.2% and 9.1% (76 and 62 compounds), respectively, present only 1 and 2 centers, corresponding to a total of 55.6% (Figure 5B). With considerably more complex structures, 6.8% (46 compounds) present more than 10 stereogenic centers.

### 3.5. Chemical Diversity

**Figure 6:**
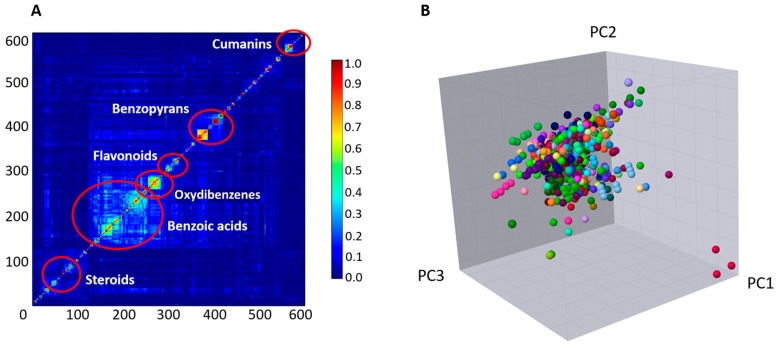
Chemical diversity analysis of the dataset: (**A**) structural similarity chart (centroid clustered) indicating the most important regions of similarity (from dark blue to dark red, respectively, 0% to 100% similarity), (**B**) 3D PCA showing the chemical diversity of the NPs with their corresponding source in distinct colors. The first three components capture 94.8% of the total variance.

The chemical diversity of the dataset was assessed using a similarity chart with descriptors based on the Tanimoto coefficient and the molecular fingerprint implemented in Canvas (see Section 2) (Figure 6A) [17]. As can be seen, the overall similarity is below 30%, indicating considerable structural diversity in the dataset. The chart displays the six most important regions of similarity (red circles), representing the main classes of compounds: cumanins, steroids, flavonoids, oxydibenzenes, benzoic acids, and benzopyrans. Benzoic acids occupy the largest portion, while oxydibenzenes exhibit the highest intra-class similarity. The chemical diversity was also evaluated by a three-dimensional principal component analysis (3D PCA) to reduce the dimensionality of the dataset, including the removal of descriptors that are highly correlated, while preserving as much of the relevant information as possible (Figure 6B) [22,23]. The distinct colors show the heterogeneity of the compounds in terms of their sources. According to the PCA results, for example, the regions of the plot in dark red (fungal isolates), light green (*Physalis minima*), pink (*Salileptolyngbya* sp.), and purple (*Arrabidaea brachypoda*) were found to be structurally correlated, despite their diverse sources.

### 3.6. Property Associations

MW is one of the most important drug-like properties (Lipinski limit of 500 Da), as small-molecule drugs (organic compounds with low MW) have been the mainstay of the pharmaceutical industry for many decades. Most small molecules can be administered orally, and they can pass through cell membranes to reach intracellular targets. Lipophilicity, represented by the partition coefficient (*p*), which is defined as the tendency of a neutral compound to dissolve in an immiscible biphasic system of lipids and water, is a key physicochemical property in medicinal chemistry [24]. The calculated descriptors of the logarithm *p* (clogP) are fundamental for predicting the permeability and absorption of bioactive compounds. 

**Figure 7:**
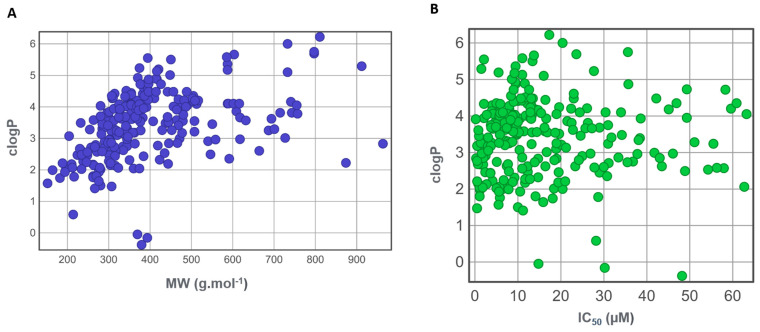
Scatter plots of associations between lipophilicity (clogP) and molecular weight (MW) and biological activity (IC_50_): (**A**) clogP versus MW for the entire dataset and (**B**) clogP versus IC_50_ values for a subset of 243 compounds with anti-*T. cruzi* activity.

Drug candidates with higher MW and lipophilicity show poor solubility and bioavailability, leading to other problems such as challenges with metabolism, permeability, or interactions with other drugs. Given the importance of these descriptors for this unique set of NPs, their relationships were investigated using a scatter plot (Figure 7A). As can be seen, the MWs are distributed predominantly across the interval from 200 to 600 g·mol^−1^, whereas the clogP values are mostly scattered from 1 to 6. A strong correlation was observed between MWs and the corresponding regions of high (clogP > 4), intermediate (clogP = 2–4), and low lipophilicity (clogP < 2). Furthermore, the relationships between lipophilicity (clogP) and anti-*T. cruzi* potency (IC_50_ values, which refer to the half-maximal inhibitory concentration) were examined for the dataset compounds (Figure 7B). The most potent compounds (IC_50_s < 10 µM) possess low to moderate lipophilicity (clogP from 1.8 to 3.5), which corroborates previous experimental findings [25].

**Figure 8:**
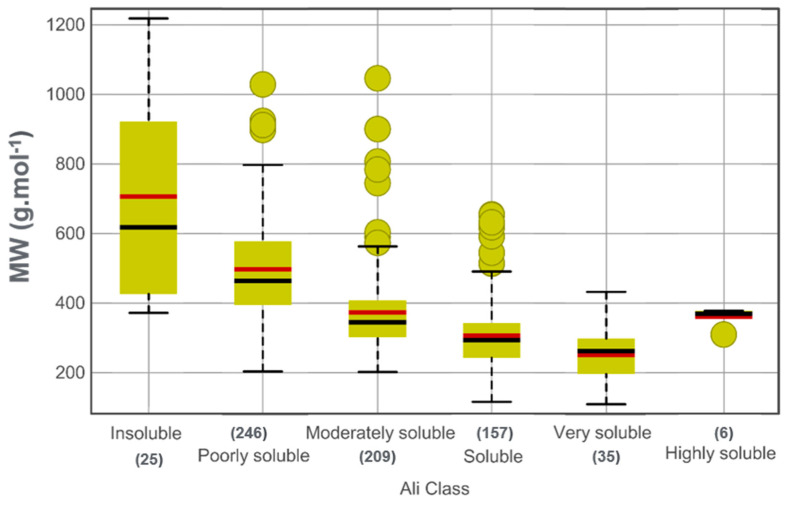
Box plot of the distribution of MW versus water solubility scores (insoluble < −10 < poorly < −6 < moderately < −4 < soluble < −2 < very < 0 < highly). Red lines indicate the mean, black lines indicate the median, and dots indicate the outliers. Dashed lines indicate the upper and lower quartiles.

Aqueous solubility is a key physicochemical property in drug discovery as it profoundly impacts bioavailability and pharmacokinetics (ADME: absorption, distribution, metabolism, and excretion) of drug candidates. It is also important in preclinical development, as the processes of hit identification, hit-to-lead, and lead optimization demand measurements of in vitro biological activity, as well as efficacy and toxicology studies in animal models [26]. The water solubility of the dataset compounds was evaluated to identify NPs with favorable oral bioavailability and pharmacokinetic characteristics (Figure 8) [14,27,28]. The results indicate that approximately 60% of the NPs have acceptable solubility (209 compounds exhibit moderate solubility, 157 are soluble, 35 are very soluble and, 6 are highly soluble). Additionally, 246 poorly water-soluble compounds and 25 insoluble compounds were identified in the dataset.

**Figure 9:**
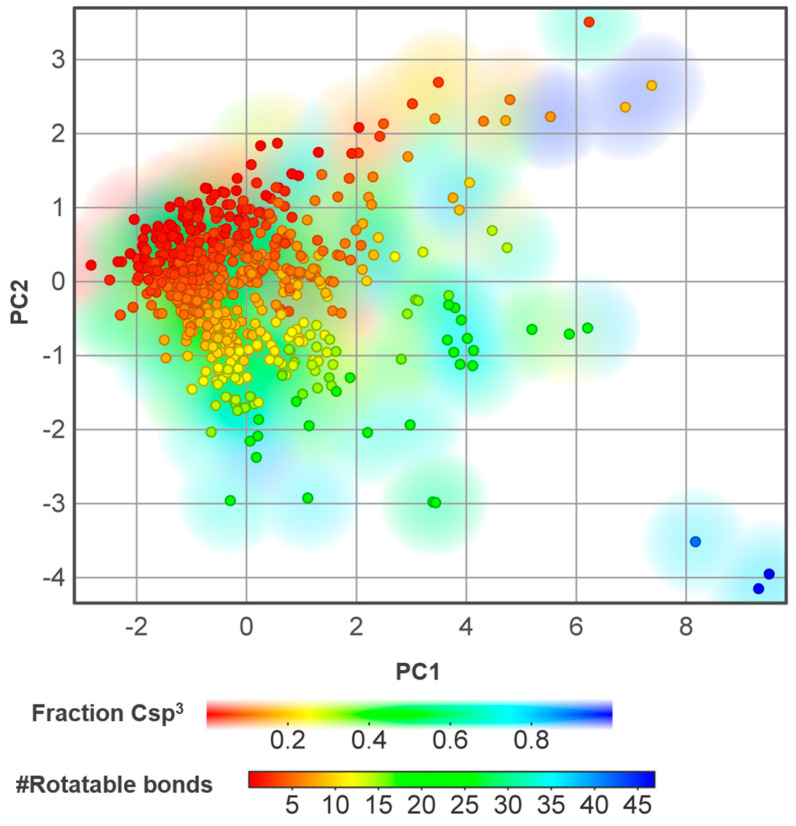
2D PCA performed using rotatable bonds (nRotB), hydrogen-bond acceptors (HBA), hydrogen-bond donors (HBD), and molecular weight (MW). Solid dot colors represent nRotB and smooth colors represent the fraction of sp^3^ hybridized carbon atoms related to the total carbon count (Csp^3^). # = number.

A PCA analysis was carried out using the dataset of NPs for the following molecular descriptors: rotatable bonds (nRotB), hydrogen-bond acceptors (HBA), hydrogen-bond donors (HBD), and MW. The contribution of hydrogen bonding capacity, the number of rotatable bonds, and the associated molecular conformational changes of small molecules are responsible for substantial differences in efficacy and pharmacokinetic properties. A molecule’s flexibility and rotatable bonds affect its ability to bind tightly to its targets, which is observed for rigid molecules with too few rotatable bonds. In addition, according to Veber’s rule of drug-likeness, compounds with more than 10 rotatable bonds are likely to exhibit low oral bioavailability [29]. In medicinal chemistry, it is important to design molecules (lead optimization stages) with an appropriate number of rotatable bonds that balance flexibility and rigidity as well as the number of HBA and HBD (hydrogen bonding capacity) for optimal binding and improved ADME characteristics. In general, the dataset compounds possess similar characteristics in terms of nRotB, HBA, HBD, and MW (Figure 9). The analysis revealed that approximately 50% of the dataset compounds have 10 or fewer rotatable bonds (nRotB, solid dots).

The increase in the degree of saturation, defined as the fraction of sp^3^ hybridized carbon atoms in relation to the total carbon count (Csp^3^), has been correlated with the probability of a compound translating from the discovery phase to clinical development [30]. Increasing Csp^3^ was found to reduce molecular planarity and packing, which, in turn, enhances water solubility. Regarding this parameter, most of the dataset compounds feature a Csp^3^ fraction > 0.25 (Figure 9). 

### 3.7. Similarity Analysis of Potent Compounds

**Figure 10:**
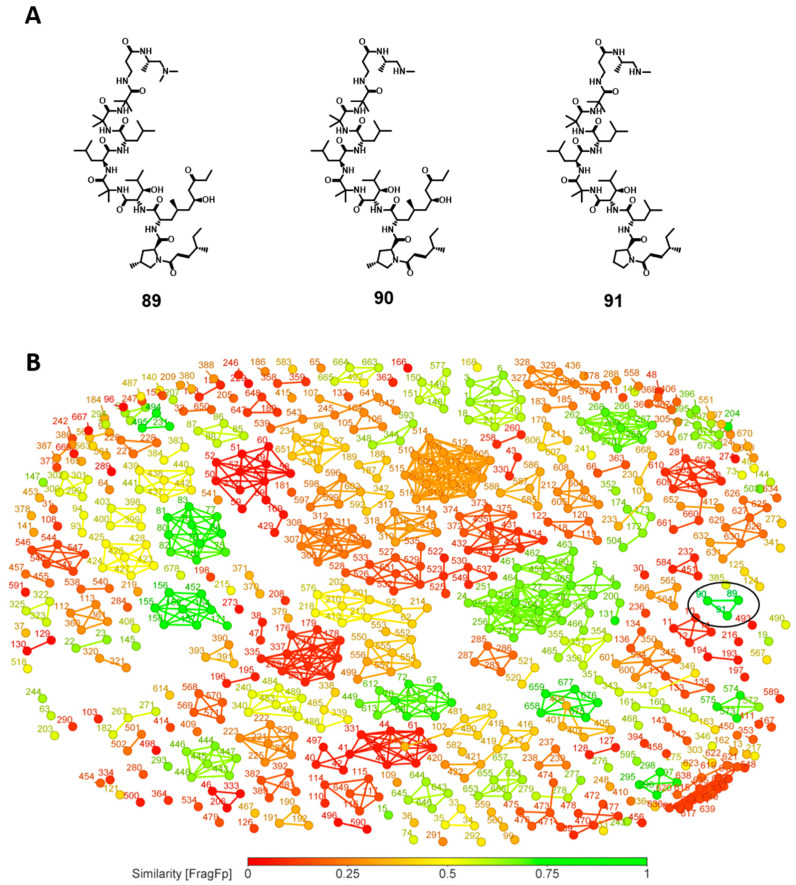
(**A**) Structures of leucinostatin A (**89**), leucinostatin B (**90**), and leucinostatin F (**91**), (**B**) similarity network for leucinostatin F (**91**).

In this study, the FragFp descriptor was selected to build similarity charts. Similarity charts show similarities between two structures using specified fragment-based descriptors. FragFp includes a dictionary with 512 substructure fragments, and the more fragments two molecules have in common, the higher is the score [31]. The most potent compound (IC_50_ = 5 nM) against the amastigote form of *T. cruzi*, leucinostatin F (**91**, Figure 10A) [32] was used as a reference to investigate the degree of structural similarity to the other compounds of the dataset (Figure 10B). In this similarity analysis, which encodes the fragments into structural fingerprints, two other leucinostatin analogues (**89** and **90**, Figure 10A) were identified with similarity greater than 95% (Figure 10B). Both leucinostatin A (**89**) and leucinostatin B (**90**) are potent anti-*T. cruzi* agents, with IC_50_ values of 7.1 nM and 12 nM, respectively.

**Figure 11:**
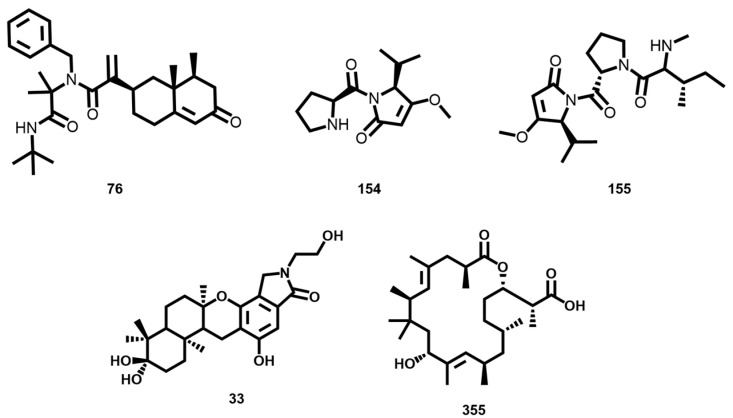
Structure of drug-like compounds **76**, **154**, **155**, **33,** and **355**.

A number of other structures, shown as nodes color-coded in green (Figure 10B), exhibited a degree of similarity to leucinostatin F of more than 70% (**91**). Nonetheless, they have the great advantage of containing active compounds with superior drug-like properties (MW ≤ 500, clogP ≤ 5, HBD ≤ 5, HBA ≤ 10, and nRotB ≤ 10). These could be explored in the design of novel antitrypanosomal drugs, including the sesquiterpene **76**, the small peptides **154** and **155**, the meroterpenoid **33** isolated from *Memnoniella dichroa*, and the polyketide strasseriolide **355** isolated from *Strasseria geniculata* (Figure 11). 

**Figure 12:**
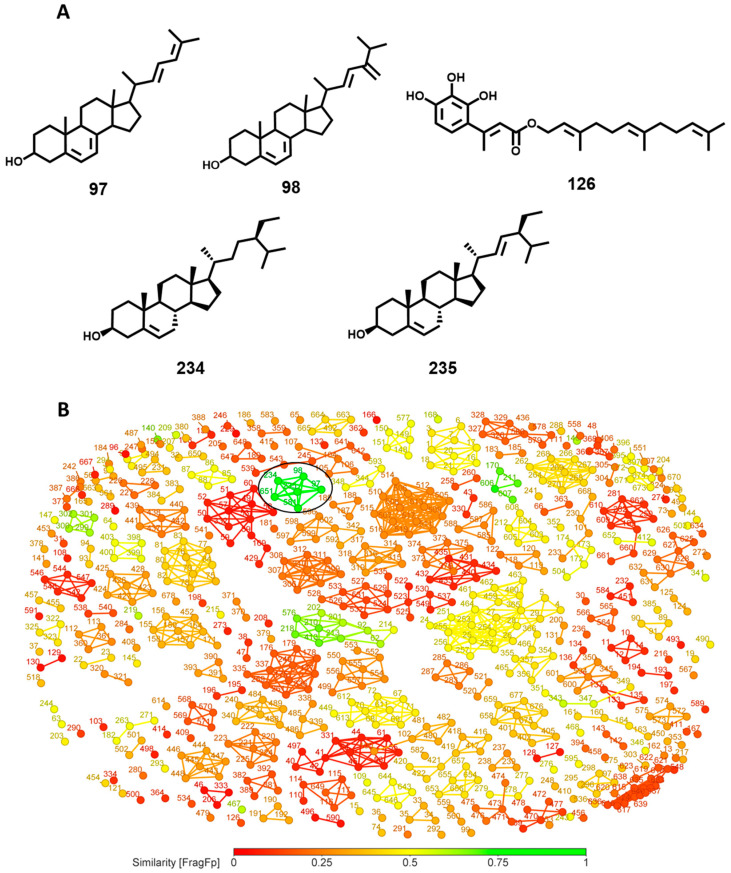
(**A**) Structure of CHT (**97**), ERGT (**98**), sesquiterpene (**126**), β-sitosterol (**234**) and stigmasterol (**235**), (**B**) similarity network for compound **97**.

Similarly, the analysis with the most potent anti-*T. brucei* compound, the steroid **97** (Figure 12A), with an IC_50_ value of 2.9 nM, revealed three compounds (**98**, **234**, and **235**) with a fingerprint-based similarity greater than 90% (Figure 12B) [33,34]. For instance, compound **98** has similarity of 96.7% and an IC_50_*^T.brucei^* of 520 nM. All compounds **97**, **98**, **234**, and **235** follow Lipinski’s and Veber’s rules. Compound **126** (IC_50_ = 12 nM) is a sesquiterpene derivative isolated from *Dorema glabrum* and it also presented a relevant IC_50_ for *L. donovani* (700 nM), demonstrating the potential of this compound for drug discovery efforts on both parasites.

**Figure 13:**
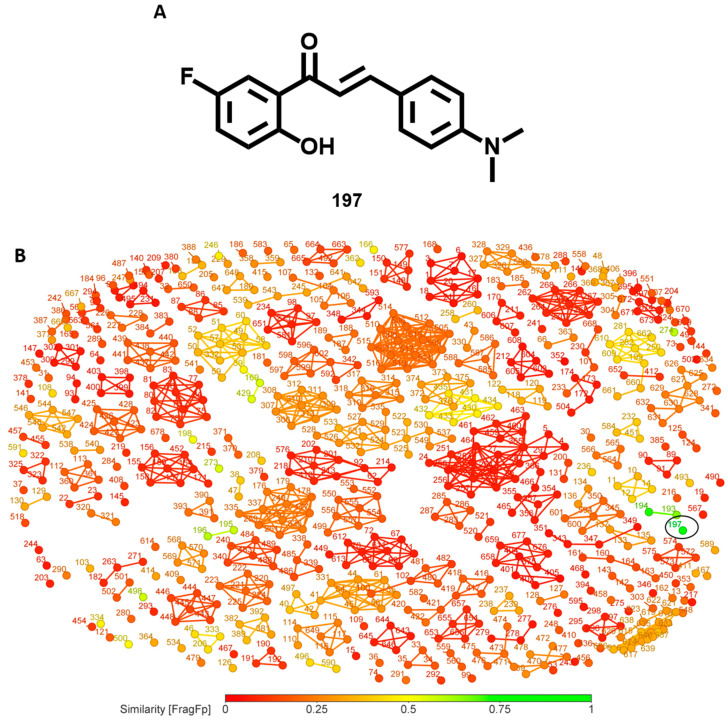
(**A**) Structure of compound **197**, a chalcone derivative, (**B**) similarity chart for compound **197**.

From a series of chalcone/flavonoid derivatives (**193**–**199**, Supporting Information) with potent activity against *Leishmania*, [35] compound **197** (Figure 13A) was selected as the reference for the similarity analysis. This compound, with an IC_50_ of 500 nM (against *L. amazonensis*), possesses rather low structural similarity (<60%) compared to the rest of the compounds in the database (Figure 13B). Given its drug-like properties and high anti-*Leishmania* potency, compound **197** could be used for similarity searches in other compound databases, providing good starting points for SAR studies.

**Figure 14:**
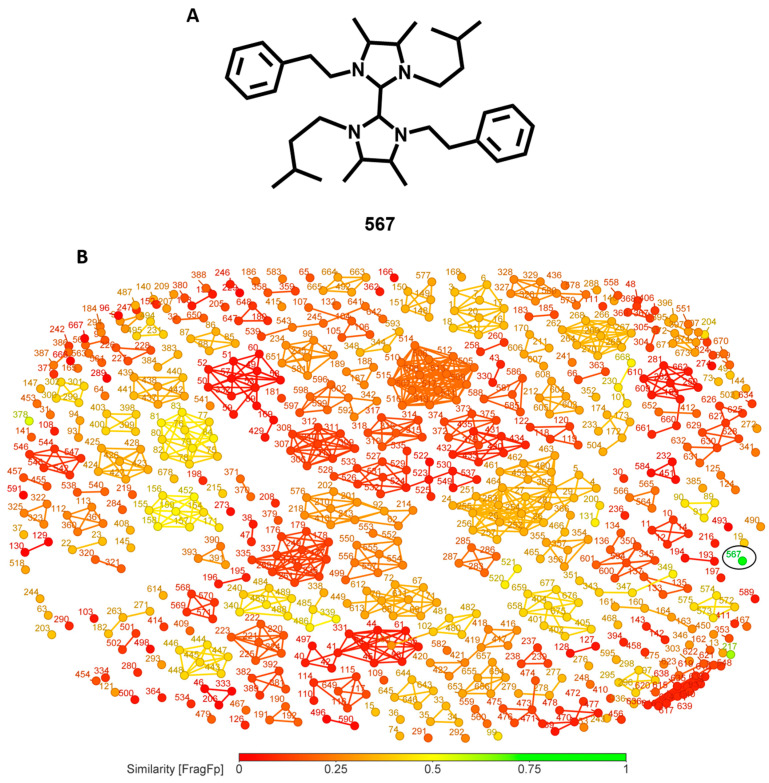
(**A**) Structure of compound **567** (**B**) similarity chart for compound **567**.

The imidazole alkaloid **567** (Figure 14A) isolated from the bacteria *Paenibacillus* sp., presented an IC_50_ of 750 nM against *L. major*. The majority of the dataset presents a similarity below 40% with this structure and no other structure in the dataset was linked in the similarity cluster (Figure 14B). Thus, compound **567** has a promising potential for SAR exploration given that no analogues were identified and tested for *L. major*.

**Figure 15:**
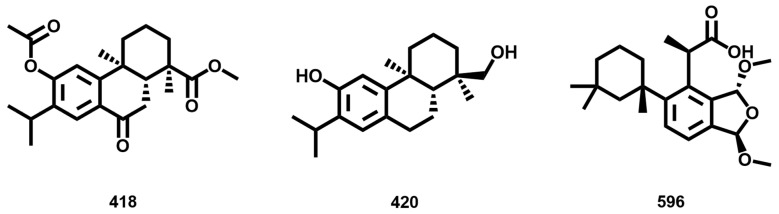
Structure of drug-like terpenoids **418**, **420,** and **596**.

Both diterpenes **418** and **420** (Figure 15) extracted from *Abies* genus showed IC_50_ values of 700 nM against *L. infantum*. The diterpene **596** (Figure 15), extracted from the marine species *Dendrilla antarctica* has an IC_50_ of 800 nM against *L. donovani*. Similarity with most of the dataset compounds is below 50% for these structures, which can represent suitable starting points for future SAR exploration.

## 4. Conclusions

The development of new bioactive molecules is facilitated by a deeper understanding of the relevant chemical space, which can improve the success rate of drug discovery efforts. Chagas disease, HAT, and leishmaniasis are NTDs for which innovation is urgently needed for the next generation of drugs. In this work, compounds from diverse natural sources have been collected through an in-depth survey of the recent literature. A chemoinformatic analysis of the 678 compounds identified several promising hits and lead candidates for anti-trypanosomatid drug discovery. Plants, the primary source of NPs explored in drug research, were the most abundant source of bioactive compounds. The secondary metabolites that were investigated comprise six major regions of structural similarity: benzopyrans, oxydibenzenes, flavonoids, steroids, benzoic acids, and cumanin derivatives. This finding shows that a few chemical classes can be privileged structural scaffolds for anti-trypanosomatid drug discovery. The steroid metabolic class was the most promising for both anti-*Trypanosoma* and anti-*Leishmania* activity. Peptides also showed promising anti-*Trypanosoma* activity, however, it was underexplored for *Leishmania*. Studies involving NPs with anti-*T. cruzi* and anti-*Leishmania* properties are more prevalent than those with *T. brucei* activity. These data reveal a tendency of the research community toward trypanosomatid diseases that are widely present across different regions of the world. Considering the lack of innovation in the NTD pharmaceutical pipeline, the results reported in this work provide valuable information to guide further NP-based drug discovery efforts on trypanosomatid diseases.

## Figures and Tables

**Table 1:** Keywords used for the literature search and the resulting number of papers.

**SciFinder-n Search**			
**Search**	**Abstract/Keywords**	**Publication Year**	**Number of Papers**
Natural products	Trypanosomatida	2019–2024	20
Natural products	Trypanosoma	2019–2024	240
Natural products	Leishmania	2019–2024	329
Natural products	Leishmaniasis	2019–2024	252
Natural products	Chagas	2019–2024	120
Natural products	Neglected Tropical Diseases	2019–2024	119
Secondary metabolites	Trypanosoma	2019–2024	36
Secondary metabolites	Leishmania	2019–2024	66
Secondary metabolites	Trypanosomatida	2019–2024	2
Secondary metabolites	Leishmaniasis	2019–2024	42
Secondary metabolites	Chagas	2019–2024	9
Secondary metabolites	Neglected Tropical Diseases	2019–2024	25
**Web of Science Search**			
**Topic**	**Topic**	**Year Published**	**Number of Papers**
Natural products	Trypanosomatida	2019–2024	13
Natural products	Trypanosoma	2019–2024	234
Natural products	Leishmania	2019–2024	311
Natural products	Leishmaniasis	2019–2024	272
Natural products	Chagas	2019–2024	113
Natural products	Neglected Tropical Diseases	2019–2024	89
Secondary metabolites	Trypanosoma	2019–2024	50
Secondary metabolites	Leishmania	2019–2024	65
Secondary metabolites	Trypanosomatida	2019–2024	2
Secondary metabolites	Leishmaniasis	2019–2024	54
Secondary metabolites	Chagas	2019–2024	16
Secondary metabolites	Neglected Tropical Diseases	2019–2024	18

## Data Availability

All data generated in this study can be found in the Supporting Information.

## Supplementary Materials

The following supporting information can be downloaded at: https://www.mdpi.com/article/10.3390/biom14081033/s1.
